# Structure and Photocatalytic Properties of Mn-Doped TiO_2_ Loaded on Wood-Based Activated Carbon Fiber Composites

**DOI:** 10.3390/ma10060631

**Published:** 2017-06-09

**Authors:** Xiaojun Ma, Wanru Zhou, Yin Chen

**Affiliations:** Department of Wood Science and Technology, Tianjin University of Science & Technology, Tianjin 300222, China; zhwanru@mail.tust.cn (W.Z.); chenyin880828@163.com (Y.C.)

**Keywords:** Mn-doped TiO_2_, activated carbon fibers, wood, photocatalyst, characterization

## Abstract

Mn-doped TiO_2_ loaded on wood-based activated carbon fiber (Mn/TiO_2_-WACF) was prepared by sol–gel and impregnation method using MnSO_4_·H_2_O as manganese source. The structure of Mn/TiO_2_–WACF was characterized by SEM, XRD, FTIR, N_2_ adsorption and UV–Vis, and its photocatalytic activity for methylene blue degradation was investigated. Results show that Mn-doped TiO_2_ were loaded on the surface of wood-based activated carbon fiber with high-development pore structures. The crystallite sizes of Mn-doped TiO_2_ in composites were smaller than that of the undoped samples. With an increase of Mn doping content, Ti–O bending vibration intensity of Mn/TiO_2_–WACF increased and then decreased. Moreover, Ti–O–Ti and Ti–O–Mn absorption peaks increased upon doping of Mn. Mn/TiO_2_–WACF with low specific surface area, and pore volume was improved at 3.5–6.0 nm of mesopore distributions due to the Mn-doped TiO_2_ load. In addition, the UV–Vis showed that Mn/TiO_2_–WACF (photodegradation rate of 96%) has higher photocatalytic activity than the undoped samples for methylene blue degradation under visible light irradiation.

## 1. Introduction

As a well-known photocatalyst, TiO_2_ has attracted lots of interest over the past decades due to its chemical stability, thermal stability, high efficiency, nontoxicity, and low cost [1]. It is extensively applied to the purification of air, bactericidal action, anti-fog, self-cleaning, and degradation of organic pollutant compounds in wastewater. However, the band gap of TiO_2_ photocatalyst is 3.2 eV, therefore only under ultraviolet (UV) excitation light this semiconductor exhibits catalytic, and the ultraviolet content of sunlight has only 3% to 5%, which limits the use of solar energy. Electrons and holes easily recombine on the surface and interior of TiO_2_ particles, which reduces the photocatalytic activity of TiO_2_ [2,3,4]. In addition, the degradation rate of the suspension-type TiO_2_ photocatalyst is slower when the concentration of target pollutant is low. On the other hand, TiO_2_ powder is difficult to be recycled, as the material gets easily inactivated and coagulates, which limits the application of nano-TiO_2_ [5,6]. Therefore, the most important and challenging issue is to develop efficient visible light responsive photocatalysts by the modification of TiO_2_ and curing.

At present, the main surface modifications used with TiO_2_ are compound semiconductor method [7,8,9], ion doping method [10,11,12,13] and precious metal deposition method [14,15], among which, ion doping of transition metal is an effective route. The studies suggest that a small amount of metal-ion-doped TiO_2_ can make TiO_2_ become potential wells to capture photogenerated electron–hole pairs; thus, the photo-generated electrons and holes are difficult to complex [16,17,18,19]. The focus of curing research is the choice of carrier and fixed process. Inorganic materials mainly used as carrier include glass, metal, ceramics, activated carbon and molecular sieves. Among them, the activated carbon fiber has become the main carrier of photocatalytic oxidation technology due to its good adsorption performance and photocatalytic synergistic effect [20]. With the growing awareness of environmental protection and the shortage of fossil resources, the sustainable development of biomass activated carbon fiber has gradually became an alternative photocatalyst carrier material and has shown excellent performance [21,22,23].

In the present study, using activated carbon fiber (wood-based activated carbon fiber, WACF) from liquefied wood as a carrier and Mn-doped TiO_2_ as photocatalyst, the photocatalytic composite materials (Mn/TiO_2_–WACF) were prepared by the sol-gel and impregnation method. The effects of different Mn doping ratios on the microcrystalline structure, specific surface area and pore size distribution of the composites were investigated in detail. Additionally, the photocatalytic degradation of photocatalytic composite for methylene blue (MB) under visible light conditions is also discussed.

## 2. Experimental

### 2.1. Chemicals

For the preparation of undoped and Mn-doped TiO_2_ photocatalytic composite materials, the materials used were ethanol (molecular mass (M) = 46.07, C_2_H_6_O), tetrabutyl titanate (M = 340.32, Ti(OC_4_H_9_)_4_), Manganese sulfate (M = 169.6, MnSO4·H_2_O) and aceticacid (M = 60.05, C_2_H_4_O_2_). For photocatalytic degradation, methylene blue dye; MB (M = 319.85, C_16_H_18_N_3_SCl) was used. All chemicals used were analytical reagent (AR) grade from Tianjin Jiangtian Chemical Co. Ltd. (Tianjin, China) and used without further purification.

### 2.2. Samples

10.2 g of Ti(OC_4_H_9_)_4_ was slowly poured into 90 mL of ethanol, and stirred with a magnetic stirrer for 30 min until colorless and transparent. Meanwhile, 3 mL of MnSO_4_·H_2_O solution (17 g/L) was added to 2.2 mL of distilled water, 2 mL of acetic acid, and 60 mL of ethanol to from another solution. Then, the latter solution was slowly poured into the former solution under vigorous stirring until completely dissolved. Finally, the mixed solution was placed in a thermostatic water bath at 40 °C for 2 h to obtain a yellowish emulsion colloid solution consisting of Mn–TiO_2_ gel.

WACF (0.2 g) [24] was evenly immersed into the colloid solution. The solution was taken out after vibration and left to stand for 30 min. Then, the solution was placed into a vacuum tube-furnace for drying process under 105 °C for 1 h. After heat treatment at 450 °C for 30 min under different calcination temperatures through N_2_ (flow rate was 100 mL/min), Mn/TiO_2_–WACF photocatalysis composite material was prepared. According to the molar ratio of Mn to Ti: 0:1, 1:600, 1:300, 1:100, and 1:50, the prepared samples were labeled as Ti–WACF, Mn/600Ti–WACF, Mn/300Ti–WACF, Mn/100Ti–WACF, and Mn/50Ti–WACF, respectively.

### 2.3. Measurements

#### 2.3.1. Scanning Electron Microscopy Analysis

JSM-7500F cold-field-emission scanning microscope (resolution at 3.0 nm and acceleration voltage at 1 kV) produced by JEOL (Tokyo, Japan) was adopted for appearance and morphology diagrams of the samples. After Mn/TiO_2_–WACF photocatalysis, composite material samples were dried and bonded on the sample table to conduct surface vacuum metal spraying and observe their surface morphologies.

#### 2.3.2. XRD Analysis

D/max2500 X-ray diffraction (XRD) was obtained on a RIGAKU instrument (Tokyo, Japan). Cu Kα X-ray was used, tube voltage was 40 kV, tube current was 100 mA, scanning angle scope 2*θ* was 20°–80°, and scanning speed was 8° min^−1^.

According to the Scherrer formula:

(1) $$D=\frac{K_{1}\lambda }{\beta_{1/2}\cos\theta }$$

The average crystallite size *D* (nm) of TiO_2_ in photocatalysis samples is calculated. *K*_1_ is the shape factor of the crystalline with a value of 0.89, *λ* is the wavelength of X-ray with a value of 0.154 nm, *β*_1/2_ is full width at half maximum of diffraction peak (rad), and *θ* is the diffraction angle (°).

#### 2.3.3. FT-IR Analysis

Nicolet 6700 Fourier transform infrared (FT-IR) spectrometer (Thermo Electron Corporation, Waltham, MA, USA) was used to analyze the samples. Samples were tested by 1:300 KBr disc technique. Absorbance spectra were acquired at 4 cm^−1^ resolution and signal-averaged over 32 scans in the scanning range of 400–4000 cm^−1^.

#### 2.3.4. Specific Surface Area and Aperture Analysis

The surface area and the porosity of the samples were determined by N_2_ adsorption–desorption isotherm measured at 77 K in a Micromeritics ASAP-2020 apparatus (Micromeritics Instrument Corporation, Norcross, GA, USA). Before analysis, the samples were degassed at 350 °C for 2 h. The specific surface area (S_BET_) was calculated by the Brunauer–Emmett–Teller (BET) method using N_2_ adsorption isotherm data. The total pore volume (V_tot_) was evaluated by converting the amount of N_2_ adsorbed at a relative pressure of 0.995 to the volume of liquid adsorbate. The micropore area (S_micro_) and micropore volume (V_micro_) were obtained by t-plot method. The mesopore area (S_meso_) and mesopore volume (V_meso_) were calculated by Barrett–Joyner–Halenda method. Pore size distributions were calculated using Density Functional Theory (DFT) Plus Software (provided by Micromeritics Instrument Corporation, Norcross, GA, USA), which is based on the calculated adsorption isotherms for pores of different sizes. This program performs an inversion of the integral equation for the overall adsorption isotherm with respect to pore size distributions.

#### 2.3.5. Photocatalysis Performance Test

33 mg of methylene blue was dissolved in 1000 mL of distilled water at 60 °C and placed in a volumetric flask. Mn/TiO_2_–WACF (10 mg) prepared under different calcination temperatures were accurately weighted and placed in a beaker with 100 mL of MB solution. The solutions were placed into a black case and magnetically stirrer for 40 min until adsorption equilibrium. A spectrophotometer (UV-1600 model produced by Shanghai MAPADA Instrument Co. Ltd. (Shanghai, China)) was used to measure and record absorbance *A*_0_ in preliminary test at 665 nm. Optical filters (transmission wavelength 400–800 nm) were covered on beakers filled with samples. Under the conditions of illumination and magnetic stirring, their absorbance was measured with a spectrophotometer every 40 min, and the decolorization ratio *D* was calculated according to Equation (2):

(2) $$D=\frac{A_{0}-A}{A_{0}}\;\times 100\%$$

where *A*_0_ is the solution absorbance before illuminance, and *A* is the solution absorbance at a certain time.

## 3. Results and Discussion

### 3.1. Morphological Characteristics

**Figure 1:**
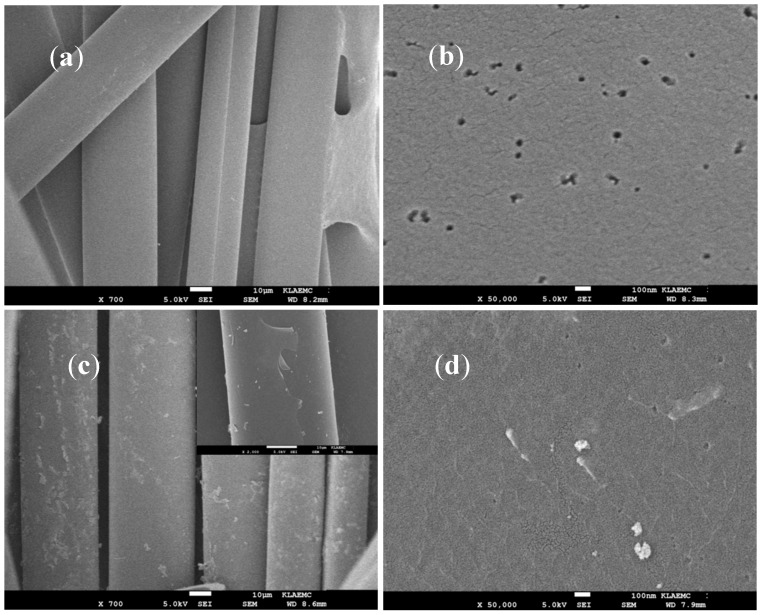
Scanning electron micrographies of the surface of wood-based activated carbon fiber (WACF) (**a**,**b**) and Mn/TiO_2_–WACF (**c**,**d**).

SEM micrographies of the WACF are shown in Figure 1a, the surface of Mn/Ti–WACF had a layer of Mn-doped nano-TiO_2_ film with uniform thickness (Figure 1c). Mn-doped nano-TiO_2_ films were loaded on the surface of WACF, but some tilted and shed in the edges, mainly due to the inconsistency of the fiber and modified photocatalyst shrinkage under high-temperature calcination. In addition, bare base materials existed on the surface of Mn/Ti–WACF (Figure 1d), which had well-reserved abundant pore structures and provided advantages for subsequent adsorption and photocatalytic reaction.

### 3.2. XRD Analysis

**Figure 2:**
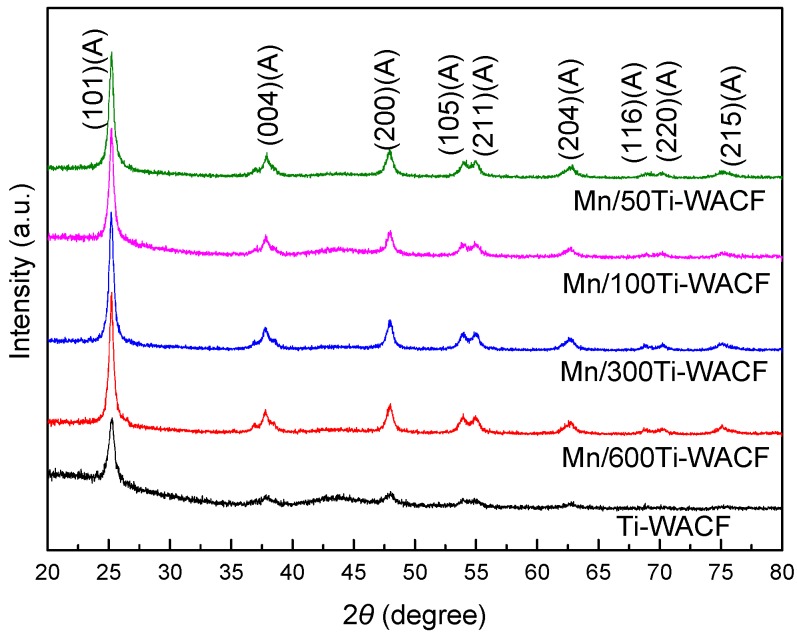
XRD diffractograms of loaded materials with different Mn doping concentrations.

Figure 2 shows an X-ray diffractograms spectrum of the various samples. The diffraction peaks of anatase TiO_2_ (101) (at 2*θ* = 25.22°) and (004) (at 2*θ* = 37.93°) were not cancelled after Mn doping, and no diffraction peaks associated with rutile and brookite (the crystal structure of TiO_2_ was consistent with that of undoped Mn) was observed, suggesting that the crystal structure of TiO_2_ was not affected by Mn doping. In addition, the intensity of these peaks decreased, and the main peak position shifted slightly with increasing Mn doping concentration, indicative of a decrease in crystallization of TiO_2_. This phenomenon also reveals that Mn ions successfully enter into the TiO_2_ crystal lattice, replacing part of the Ti ions, destroying the symmetry of the crystal structure of TiO_2_, causing crystal lattice disorder and increasing distortion, which results in defects in the lattice [25,26]. The full-width half maximum increased with increasing Mn doping concentrations, which indicates that doping Mn effectively controlled the recrystallization or grain size of the TiO_2_ sintering during calcination. No impurity peak was associated with the Mn element in the XRD crystalline spectra of the samples due to scarce Mn doping.

Table 1 presents the average crystallite size of modified nano-TiO_2_, which were calculated based on Scherrer formula. The grain size of nano-TiO_2_ gradually increases with increasing Mn doping concentrations. The crystallite sizes (25.4–27.8 nm) of TiO_2_ after doping Mn were obviously smaller than that of undoped Mn, indicating that Mn doping can inhibit the growth and agglomeration of TiO_2_ grains. This small size promotes TiO_2_ to evenly and firmly wrap on the surface of WACF, providing a superior condition for the photocatalytic reaction.

### 3.3. FTIR Spectroscopy

**Figure 3:**
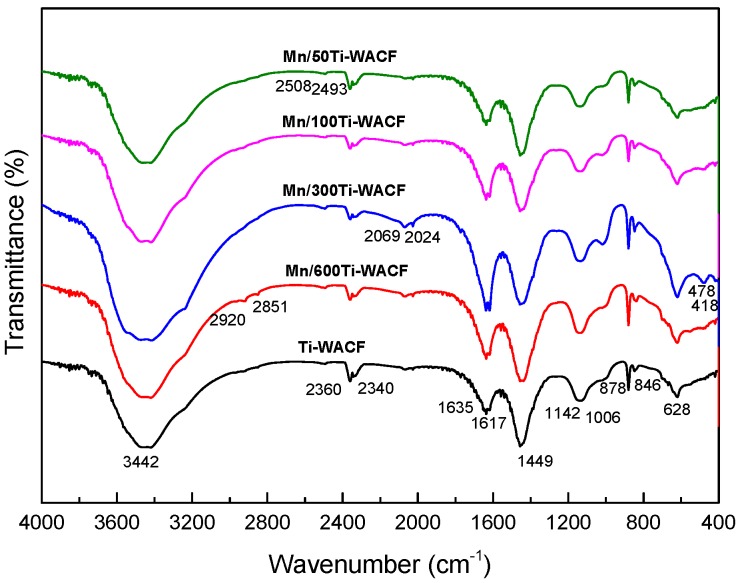
FTIR spectra of Mn/TiO_2_–WACF samples with different Mn doping concentrations.

FTIR spectra of TiO_2_ doped with different Mn doping concentration on WACF are shown in Figure 3. Stretching and bending vibration of hydroxyl groups of all loaded materials were in the range of 3200–3600 cm^−1^ and 1617–1635 cm^−1^, and the stretching vibration band intensity of Mn-doped samples was clearly stronger than that of the undoped sample, indicating that TiO_2_ had a stronger adsorption with Mn-doping. The absorption bands of loaded materials at 1444 cm^−1^ were associated to the stretching of C–H bonds and C–O–Ti. A smaller amount of Mn doping caused intensity enhancement of absorption bands, indicating that less Mn doping promotes TiO_2_ loading on the WACF surface, as observed in the XRD measurements. Moreover, the absorption bands of all loaded materials in the vicinity of 618 cm^−1^ belonged to the characteristic absorption peaks of anatase TiO_2_ (consistent with the XRD test results), which represented the bending vibration of Ti–O, and the peak intensity was bounded by the Mn/Ti molar ratio of 1:300. With the increase of Mn doping amount, Ti–O bending vibration intensity would initially increase and then decrease, and the intensity of Mn-doped materials was bigger than that of pure TiO_2_. The latter had only Ti–O bending vibration, and the former has Ti–O and Ti–O–Mn bond; this finding indicates that Mn doping was good for TiO_2_ loading on WACF and for the formation of anatase TiO_2_. The absorption band of the undoped and doped samples is over the range of 846–878 cm^−1^; the former had a weak band and only Ti–O–Ti stretching vibration. The latter had stronger intensity bands due to the dual role of Ti–O–Ti and Ti–O–Mn [27].

In addition, the samples showed a weak absorption band in the range of 2340–2360 cm^−1^ and 1006–1142 cm^−1^, representing the P–H stretching vibration and the P–O–C bond; this finding was mainly due to the addition of the phosphoric acid catalyst during the liquefaction of the wood.

### 3.4. Specific Surface Area, Pore Volume and Aperture Analysis

**Figure 4:**
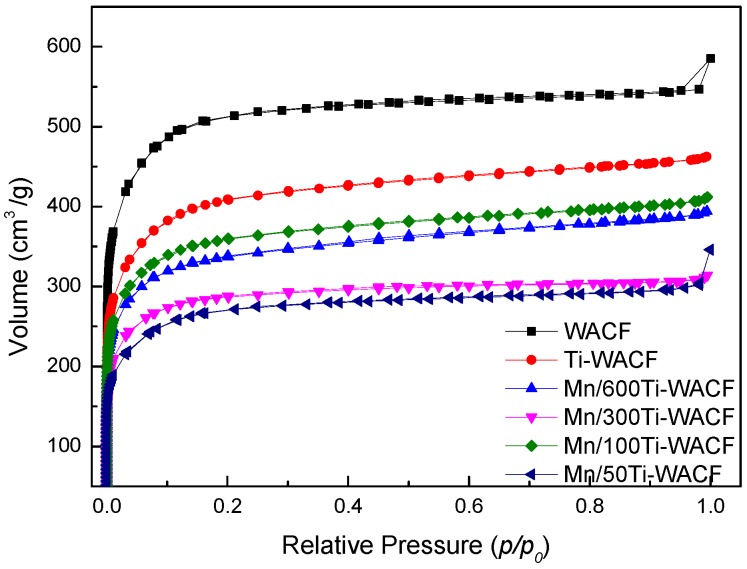
N_2_ adsorption/desorption isotherm of WACF and Mn/TiO_2_–WACF samples.

The adsorption/desorption isotherms of WACF and Mn/TiO_2_–WACF with different Mn doping concentrations are shown in Figure 4. The figure shows that all samples belong to the typical I-type adsorption isotherm (Langmuir isotherm), and the pore structure is dominated by micropores. The curves of WACF and Mn/50Ti–WACF experienced obvious hangover boosting phenomena, and they belonged to I-B type material; the other materials belonged to I-A type material. The N_2_ adsorption capacity of unloaded WACF sample was higher than that of Mn/TiO_2_–WACF sample. The hysteresis of the former adsorption and desorption processes was more obvious than the latter, indicating that the former had larger mesopores and macropores than the latter. This finding indicates that the photocatalyst was successfully loaded onto the fiber surface or filled with the pores of the load material. Meanwhile, N_2_ adsorption quantity of Mn-doped material was lower than that of the undoped material, indicating that the load rate of TiO_2_ became higher after Mn doping.

**Figure 5:**
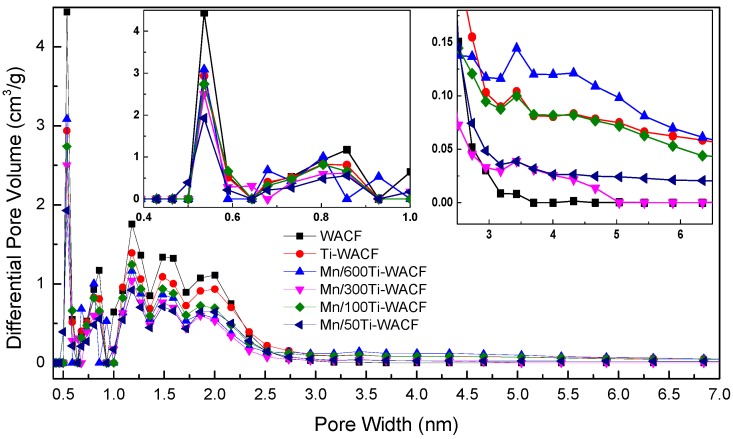
Pore size distribution (density functional theory method) of WACF and Mn/TiO_2_–WACF.

The pore diameter distribution (DFT method) of WACF and Mn/TiO_2_–WACF are shown in Figure 5. The curve shows that the pore size of WACF was mainly distributed between 0.4 nm and 3.5 nm, and that Mn/TiO_2_–WACF was mainly distributed between 0.4 nm and 6 nm. Therefore, the pore diameter distribution diagram of WACF and Mn/TiO_2_–WACF was similar, and no multimodal phenomenon was found. However, the Mn/TiO_2_–WACF had a small amount of mesopore between 3.5 and 6 nm, and the WACF was almost useless because the former was subjected to secondary calcination after loading the photocatalyst onto the latter surface. The latter was equivalent to the second activation by the evaporated photocatalyst to reduce, as much as possible, the TiO_2_ photocatalyst, resulting in pore diameter distribution of carrier fiber decline. In addition, the pore volume of the loaded material decreased compared with that of WACF, indicating that Mn-doped TiO_2_ photocatalyst can not only be wrapped on the fiber surface, but also it can be filled into the pores or attached to the hole wall. The pore volume of Mn-doped material was less than that of pure TiO_2_-loaded material, which again showed that Mn doping can improve the TiO_2_ loading rate. This result is consistent with the conclusion of adsorption isotherm.

Specific surface area, pore volume, and pore radius of the samples are shown in Table 2. In comparison with WACF and Ti–WACF, S_BET_, S_micro_, S_meso_, V_tot_, V_micro_ and V_meso_ of Mn/TiO_2_–WACF were reduced. The surface area and pore volume of Mn/TiO_2_–WACF were as high as 1239 and 0.628 cm^3^/g, respectively. Moreover, with the increase of Mn doping content, P_Mic_ of Mn/TiO_2_–WACF increased first and then decreased, whereas S_meso_, V_meso_ and P_Me_ decreased first and then increased, indicating that Mn doping had a certain effect on the micropores and mesopores. 

### 3.5. UV–Vis Analysis

**Figure 6:**
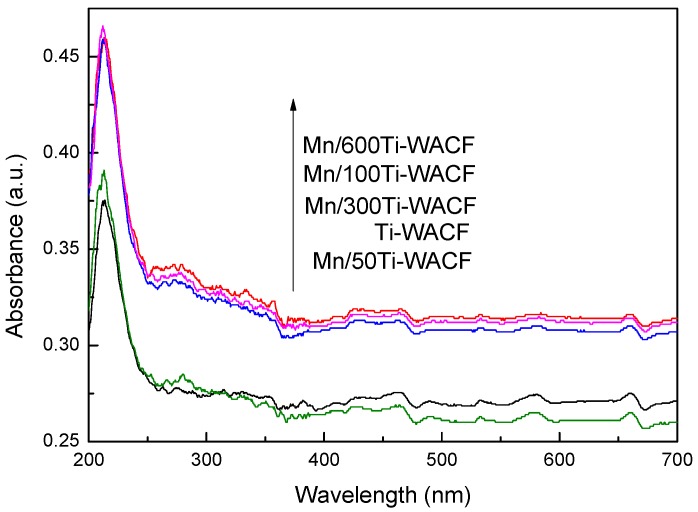
UV–Vis spectra of loaded materials with different Mn doping concentrations.

The UV–Vis spectral absorption curve of loaded materials with different Mn doping concentrations is shown in Figure 6. In the visible light region, the absorption difference of TiO_2_ material was not significant with different Mn:Ti molar ratios, i.e., 1:600, 1:300, and 1:100; these results are mainly due to the fact that the actual amount of Mn ions added was similar yet had greatly increased in comparison with pure TiO_2_-loaded materials (the maximum improvement was Mn/600Ti–WACF material). This finding indicated that Mn doping can change the light absorption properties of TiO_2_, and also showed that the effect of Mn doping was not affected by the loading process and the carrier fibers [28]. The visible light absorbance of Mn and Ti molar ratio of 1:50 was slightly lower than that of pure TiO_2_, while the Mn doping amount was excessive and some of the impurity levels provided the chance of electron–hole pairing in the photocatalytic reaction.

### 3.6. Visible Photodegradation of Methylene Blue Solution

**Figure 7:**
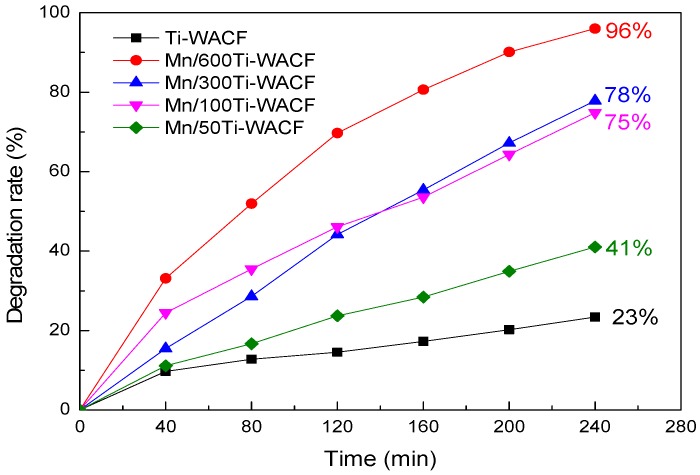
Degradation curve chart of methylene blue by samples with different Mn-doping concentrations under visible lights.

The visible light degradation curves of MB solution with varying illumination time are shown in Figure 7. The Figure 7 shows that the degradation rate of the loading material increased with the prolongation of the illumination time, and the degradation effect of the Mn-doped sample was better than that of pure TiO_2_. The degradation of Mn/600Ti–WACF samples was the most satisfactory, which was up to 96%, and 73% higher than that of TiO_2_. With the increase of Mn doping concentration, the adsorption effect of the initial stage was enhanced, and the photocatalytic effect at later stage is enhanced; thus, Mn doping can improve the adsorption performance of the TiO_2_-loaded material in the liquid environment and the concentration of target contaminants around the photocatalytic material, providing favorable conditions for subsequent photodegradation ranges.

## 4. Conclusions

Mn-doped TiO_2_ loaded on WACF (Mn/TiO_2_–WACF) was prepared by sol–gel and impregnation method. The particle sizes of TiO_2_ in Mn/TiO_2_–WACF ranged from 25.4 to 27.8 nm, and were smaller than that of the undoped samples. With the increase of Mn doping content, Ti–O bending vibration intensity of Mn/TiO_2_–WACF increased and then decreased. Moreover, Ti–O–Ti and Ti–O–Mn absorption peaks increased because of the doped Mn. The surface area and pore volume of Mn/TiO_2_–WACF were as high as 1239 m^2^/g and 0.628 cm^3^/g, respectively. In addition, Mn/TiO_2_–WACF can reach the highest photodegradation rate of 96% on MB under visible-light irradiation, which is higher by 73% than that of WACF loaded with TiO_2_. Mn/TiO_2_–WACF possesses relatively strong absorbency and photocatalytic activity after Mn doping.

## Figures and Tables

**Table 1:** Average crystallite size of nano-TiO_2_ doped with different Mn doping concentrations.

Samples	Ti–WACF	Mn/600Ti–WACF	Mn/300Ti–WACF	Mn/100Ti–WACF	Mn/50Ti–WACF
Average crystallite size	36.4	25.4	26.6	27.8	27.5

**Table 2:** Specific surface area, pore volume, and aperture parameters of WACF and Mn/TiO2–WACF.

Sample	S_BET_	S_micro_	S_meso_	V_tot_	V_micro_	V_meso_	P_Mic_^a^	P_Me_^b^	D ^c^
	(m^2^/g)	(m^2^/g)	(m^2^/g)	(cm^3^/g)	(cm^3^/g)	(cm^3^/g)	(%)	(%)	(nm)
WACF	1802	1272	530	0.875	0.581	0.294	66.4	33.6	1.94
Ti–WACF	1418	852	408	0.710	0.384	0.272	54.1	38.3	2.00
Mn/600Ti–WACF	1160	764	309	0.602	0.348	0.230	57.8	38.2	2.08
Mn/300Ti–WACF	980	691	205	0.477	0.317	0.137	66.5	28.7	1.95
Mn/100Ti–WACF	1239	803	322	0.628	0.364	0.230	58.0	36.6	2.03
Mn/50Ti–WACF	966	624	342	0.556	0.284	0.272	51.1	48.9	2.03

^a^ Ratio of the micropore volume to the total pore volume; ^b^ Ratio of the mesopore volume to the total pore volume; ^c^ Average pore diameter.
